# Significance of the Powder Metallurgy Approach and Its Processing Parameters on the Mechanical Behavior of Magnesium-Based Materials

**DOI:** 10.3390/nano15020092

**Published:** 2025-01-09

**Authors:** Sachin Kumar Sharma, Sandra Gajević, Lokesh Kumar Sharma, Dhanesh G. Mohan, Yogesh Sharma, Mladen Radojković, Blaža Stojanović

**Affiliations:** 1 Surface Science and Tribology Lab, Department of Mechanical Engineering, Shiv Nadar Institute of Eminence, Gautam Buddha Nagar 201314, India; 2 Faculty of Engineering, University of Kragujevac, SestreJanjić 6, 34000 Kragujevac, Serbia; blaza@kg.ac.rs; 3 Department of Physics, GLA University, Mathura 281406, India; lokesh.sharma@gla.ac.in; 4 School of Engineering, Faculty of Technology, University of Sunderland, Sunderland SR6 0DD, UK; dhanesh.mohan@sunderland.ac.uk; 5 Centre of Research Impact and Outcome, Chitkara University Institute of Engineering and Technology, Chitkara University, Punjab 140401, India; 6 Department of Physics, Faculty of Applied and Basic Sciences, SGT University, Gurugram 122505, India; yogesh_fosc@sgtuniversity.org; 7 Faculty of Technical Sciences, University of Priština in Kosovska Mitrovica, Kneza Miloša 7, 22202 Kosovska Mitrovica, Serbia; mladen.radojkovic@pr.ac.rs

**Keywords:** reinforcement, fabrication methodology, metal matrix composite, powder metallurgy, processing parameters

## Abstract

Magnesium-based materials, which are known for their light weight and exceptional strength-to-weight ratio, hold immense promise in the biomedical, automotive, aerospace, and military sectors. However, their inherent limitations, including low wear resistance and poor mechanical properties, have driven the development of magnesium-based metal matrix composites (Mg-MMCs). The pivotal role of powder metallurgy (PM) in fabricating Mg-MMCs was explored, enhancing their mechanical and corrosion resistance characteristics. The mechanical characteristics depend upon the fabrication methodology, composition, processing technique, and reinforcement added to the magnesium. PM is identified as the most efficient due to its ability to produce near-net shape composites with high precision, cost-effectiveness, and minimal waste. Furthermore, PM enables precise control over critical processing parameters, such as compaction pressure, sintering temperature, and particle size, which directly influence the composite’s microstructure and properties. This study highlights various reinforcements, mainly carbon nanotubes (CNTs), graphene nanoparticles (GNPs), silicon carbide (SiC), and hydroxyapatite (HAp), and their effects on improving wear, corrosion resistance, and mechanical strength. Among these, CNTs emerge as a standout reinforcement due to their ability to enhance multiple properties when used at optimal weight fractions. Further, this study delves into the interaction between reinforcement types and matrix materials, emphasizing the importance of uniform dispersion in preventing porosity and improving durability. Optimal PM conditions, such as a compaction pressure of 450 MPa, sintering temperatures between 550 and 600 °C, and sintering times of 2 h, are recommended for achieving superior mechanical performance. Emerging trends in reinforcement materials, including nanostructures and bioactive particles, are also discussed, underscoring their potential to widen the application spectrum of Mg-MMCs.

## 1. Introduction

The high strength-to-weight ratio of magnesium and its alloys makes them particularly useful in industry. However, the low formability at high temperatures reduces the strength of the Mg and its alloys [1,2]. Therefore, the metal matrix composites serve the purpose of widening the application usage of Mg-based alloys. The Mg-based MMCs involve Mg (low density) as a matrix and fibers or particles as reinforcing agents, providing high stiffness and strength at a high temperature [3]. The addition of reinforcing particles to Mg enhances mechanical behavior with high wear and corrosion resistance [4,5]. Furthermore, the beneficial aspects of MMCs include high thermal stability, good tribological properties, a controllable coefficient of thermal expansion, and superior electrical and damping properties [6,7,8]. However, the mechanical characteristics of MMC mainly depend upon the percentage and types of reinforcement, fabrication approach, matrix, fiber composition, and processing parameters [9,10,11,12]. Furthermore, severe plastic deformation and alloying strain hardening can enhance strength [13]. Concerning the beneficial aspect of Mg-MMCs, researchers are keen to incorporate a suitable fabrication technique to prepare composites. The enlisted processing techniques, i.e., friction stir processing (FSP) [14], stir casting [15], powder metallurgy (PM) [16], disintegrated melt deposition [17], and spray deposition [18], etc., are used to prepare the magnesium-based matrix composite. Compared to other MMC fabrication techniques, the powder metallurgy method has several advantages, including the ability to fabricate composites of insoluble materials [19]. PM produces nearly net-shaped products with little machining needed, and fabricated composites with high melting points produce extremely little scrap [20]. This technique controls porosity, produces self-lubricating materials, and offers good vibration and dampening characteristics [13].

**Figure 1:**
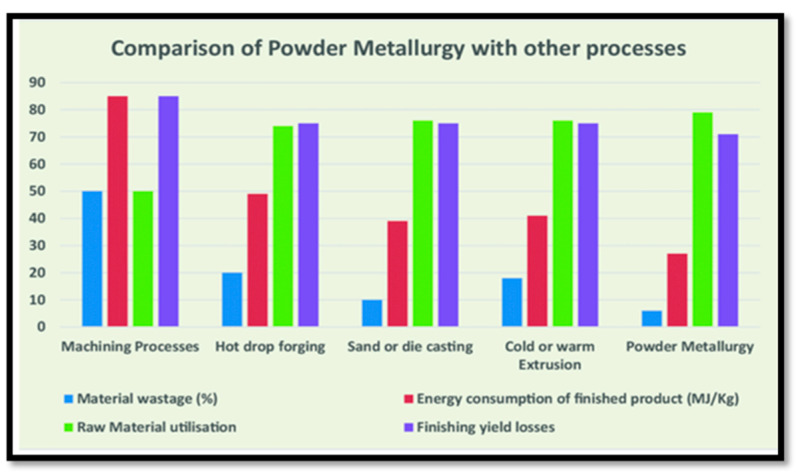
Comparative study of powder metallurgy with other processes [30].

Powder metallurgy (PM) has the aforementioned benefits and is one of the most widely utilized methods for creating MMCs. The PM approach acts as a cost-effective preparation approach [21,22,23]. Figure 1 depicts the comparative advantages of powder metallurgy over the other conventional processes. The PM technique ensures the homogeneous dispersion of reinforcing agents into the matrix and requires a lower temperature than other melting techniques [24,25,26]. PM generates complex forms with accurate sizes and shapes at a high production rate and low cost [27]. Particles in the combination of elemental or pre-alloyed powders are sintered in a furnace after being compressed in a die in the P/M process. Due to characteristics like refractoriness, high hardness, wear resistance, etc., several types of ceramic materials are frequently employed to reinforce Mg-based alloys [28,29]. The PM approach makes it simple to create components with complex dimensions and high-strength components. Magnesium matrix composites are being produced sequentially in a straightforward and economical manner by adhering to the powder metallurgy manufacturing technique.

Although composites have better mechanical characteristics than alloys, the PM approach reduces porosity, poor wetting, and interfacial energies [31,32]. The reduction in these flaws leads to an improvement in the mechanical performance of composites. In comparison to composites made using traditional methods like liquid infiltration, PM is inexpensive, is conveniently accessible, reduces the wastage of material, and achieves a better surface finish (0.80–1.20 µm) [33,34,35]. It can efficiently process complex shapes and control porosity and requires less machining, and the parts acquire good damping properties [35]. PM forms parts with combinations of materials (ceramics and metals), mainly tungsten and tungsten carbide [36]. PM provides good chemical homogeneity with higher dimensional accuracy and allows the homogenous dispersion of reinforcement into the matrix throughout [37]. Therefore, the production of metal matrix composites using powder metallurgy processes is revolutionary in both research and industrial applications. Regardless, composite materials with improved strength and hardness are created via the powder metallurgy approach. Machining, casting, hot working, and cold working processes are used to make components that fit the required dimensions. However, it is impossible to combine metals and non-metals and create components with suitable mechanical characteristics [38,39,40,41,42]. Still, the PM approach safeguards the aforementioned limitations by synthesizing and combining non-metal and metal powders in the proper ratio [43,44,45]. The blended powder is pressed into a die to obtain the desired dimensions, and sintering is used to make it tougher. Once the secondary finishing process is completed, the component has the desired characteristics, shape, and size. Further, the various processing parameters involved in the powder metallurgy approach have been discussed in detail in this review paper.

## 2. Processing Approach: Powder Metallurgy (PM)

Powder metallurgy is a solid-state approach employed in forming metal matrix composites (MMCs) that involves the blending/mixing of reinforcing and matrix powder materials. The blended powders are then compacted at a requisite pressure to obtain the green compact. Then, the sintering of the green compact is accomplished at a sintering temperature below the melting temperature to obtain the required size of the final product [46]. The PM approach involves the mixing of matrix and reinforcement powder, compaction of powder materials, and sintering of the green compact, which are discussed below.

### 2.1. Mixing of Powder Materials

The reinforcing and matrix powder materials are mixed in a ball mill to obtain the uniform dispersion of powder materials in the MMCs. During the mixing of the material powder, stainless steel balls are added to a ball mill at a ball-to-powder ratio of 10:1 [47]. The blending of the material powder is vital to retard the accumulation of powder in the composite via the uniform mixing of the powders [48]. Accumulation in the composite material can lead to a reduction in grain refinement and the development of small cracks on the surface of the composite, thereby deteriorating the properties [49]. Therefore, the reinforcement and matrix powder should be adequately mixed through ball milling to spread the reinforcing agent uniformly throughout the matrix. Thus, the mixing of powder is the crucial step of powder metallurgy.

### 2.2. Compaction of Powder Materials

After mixing the reinforcement and matrix materials, the powder is compacted through a cylindrical die by applying high pressure to obtain the appropriate shape and size of the material. The compact pressure is crucial in order to reduce the porosity and improve the strength of the composite materials [50]. Therefore, a suitable compaction pressure is required to significantly enhance the mechanical behavior of the composites and obtain a strong green compact to enable the uncomplicated handling of the material [51]. The compaction pressure is directly related to the porosity and strength of the green compact. The porosity of the green compact decreases as the compact pressure increases.

### 2.3. Sintering of Green Compact

**Figure 2:**
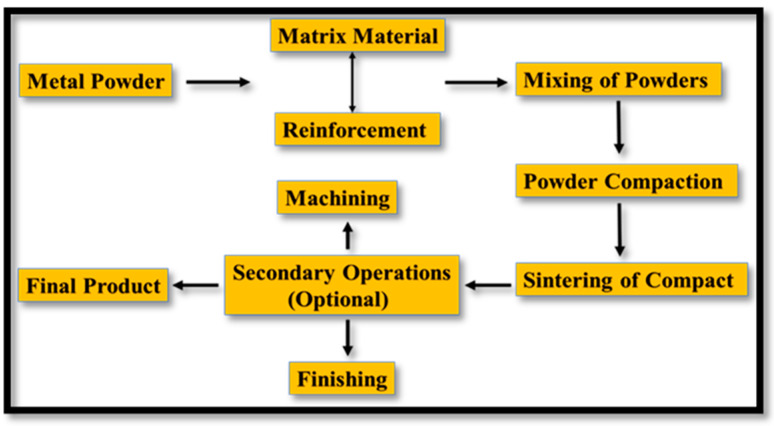
Primary processing parameters and secondary operations of powder metallurgy.

Sintering the green compact is the last and most important step of powder metallurgy, as it enhances the endurance and strength of the green compact. In the sintering process, the diffusion of the material occurs in pores that enable a chemical and physical bond at the surface interface of the reinforcing and matrix powder materials, which improves the strength of the composite material [52,53,54,55]. The research study suggested that the diffusion of the material varies with temperature and time, indicating that the sintering time and temperature determine the diffusion and expansion rate of powder materials in the composite [56,57,58]. The sintering temperature is calculated as 0.8–0.9 times the melting temperature of the material [59]. The literature study reported that a suitable sintering time also enhances the strength of the composite. Figure 2 depicts the process diagram of the powder metallurgy approach.

## 3. Processing Parameters of PM and Reinforcement

The processing parameters of PM are vital in accessing the mechanical behavior of the composite. The rate and types of reinforcement, compaction pressure, sintering time, sintering temperature, size of reinforcement, and matrix material are such processing parameters [60]. The significance of the above-mentioned parameters in terms of the microstructure and mechanical characteristics is discussed below.

### 3.1. Compaction Pressure

**Figure 3:**
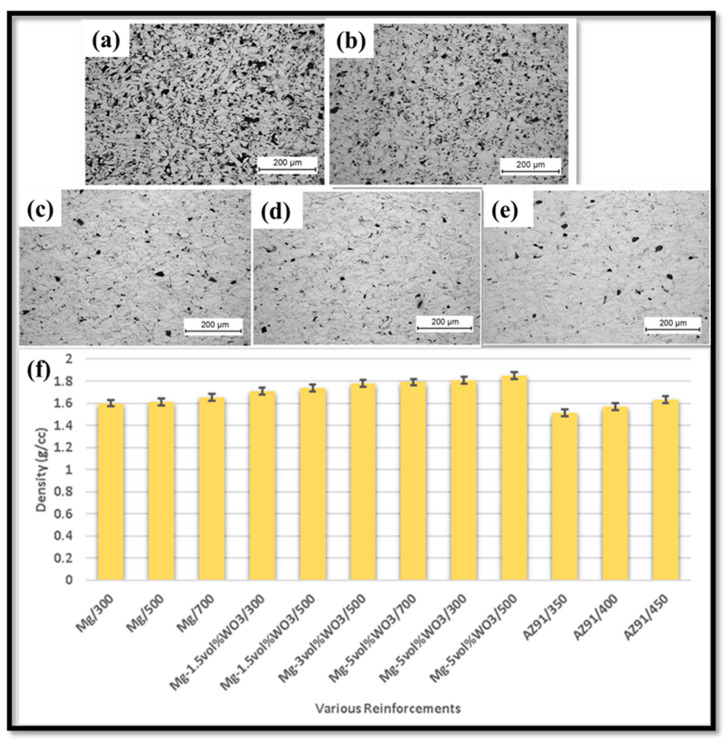
SEM image of Mg-based alloy formed at different compaction pressures: (**a**) 100 MPa, (**b**) 200 MPa, (**c**) 300 MPa, (**d**) 400 MPa, and (**e**) 500 MPa. (**f**) Variation in density of composite with compaction pressure [67].

Compaction pressure is a crucial processing parameter of PM. The porosity and density of the composites produced using PM act as a function of compaction pressure [61,62]. The high value of compaction pressure involves low porosity and high density. Compaction pressure also influences the pore’s size and the number of pores [63]. The mechanical characteristics of a material are directly dependent on porosity and density; hence, they are significantly influenced by compaction pressure [64,65]. Yusof et al. [66] investigated the effects of compaction pressure on the mechanical characteristics of a binary and ternary magnesium-based alloy that is used for biodegradable implantation. The mixture of Mg-9wt.%Zn-1wt.%Mn and Mg-10wt.%Zn alloy was mechanically alloyed in a planetary ball mill by varying the compaction pressure from 100 MPa to 600 MPa, followed by sintering at a temperature of 300 °C for 1 h. The 2 h of milling time obtained a homogenous supersaturated solid solution of Mg-9wt.%Zn-1wt.%Mn and Mg-10wt.%Zn alloy. The density of the alloying sample was determined using Archimedes’ density measurement and was found to increase with an increase in compaction pressure up to 400 MPa; however, beyond that, the density decreased. The compressive strength and hardness of the Mg-9wt.%Zn-1wt.%Mn and Mg-10wt.%Zn alloy were observed to increase up to 400 MPa, and beyond that, there exists a reduction in compressive strength and hardness of the composite. However, the compressive strength and hardness of the Mg-9wt.%Zn-1wt.%Mn (255 MPa and 72.5 Hv) alloy were higher compared to that of the Mg-10wt.%Zn (245 MPa and 66.9 Hv) alloy. The compaction pressure of 400 MPa was regarded as an optimum value that improves the mechanical behavior of composites. Brezina et al. [67] analyzed the influence of compaction pressure on the microstructure of magnesium by varying compaction pressure, i.e., 100 MPa to 500 MPa. The compaction pressure of 100 MPa had minimum plastic deformation with the highest porosity, and increasing the compaction pressure resulted in lower porosity and maximum plastic deformation. The compaction pressure between 300 MPa and 500 MPa obtained similar microstructural characteristics (Figure 3a–e).

The correlation between microhardness and compaction pressure is primarily influenced by the mechanisms of densification and microstructural changes that occur during the compaction and sintering processes [63]. The impact of compaction pressure on microhardness is illustrated as follows:

1. *Increased Densification:* Higher compaction pressure leads to greater densification of the composite material. As the particles are forced closer together, the contact area between them increases, facilitating better bonding during subsequent sintering [65]. This densification effectively reduces porosity, which is critical for enhancing mechanical properties, including microhardness.
2. *Grain Refinement:* The application of a higher compaction pressure can also promote grain refinement in Mg-based composites. Smaller grain sizes typically result in higher hardness values due to the Hall–Petch effect, where finer grains impede dislocation movement, thereby increasing resistance to deformation [66].
3. *Interfacial Bonding:* Increased compaction pressure improves the interfacial bonding between the matrix and reinforcing phases present in the composite. This enhanced bonding contributes to improved load transfer during mechanical testing, resulting in higher microhardness [67].

Practically all desirable characteristics of a material, including shape, size, porosity, hardness, density, and other mechanical and thermal properties, could be controlled in the process of compaction [68,69]. Depending on punch movement and type of die (single-piece or split), powder compaction can be performed in a variety of ways [70]. There are two types of compactions, uniaxial and multiaxial, which depend on the punch movement [71]. Single-action uniaxial compaction signifies the movement of the upper punch while the lower punch stays stationary [72]. Double-action uniaxial compaction is a process that involves the significant movement of the lower and upper punches [73]. Multiaxial compaction is when the powder is compressed from multiple sides simultaneously. When the powder is subjected to isostatic pressing, pressure is applied across all axes, suggesting multiaxial compression [74]. Powder compaction pressure, pressure type (uniaxial or isostatic), kind of matrix material, and type of reinforcing agent all affect the properties of a powder metallurgy green compact (metallic, carbonaceous, or ceramic) [75]. Table 1 details the comparative study of powder compaction via various dies. Rahmani et al. [76] analyzed the effects of compaction pressure on the tribological and mechanical characteristics of magnesium–tungsten trioxide (Mg-WO_3_) at variable compaction pressure, i.e., 300 MPa, 500 MPa, and 700 MPa. The relative density and hardness of the alloy were maximal at 700 MPa due to the continuous reduction in porosity. Kumar et al. [77] analyzed the effects of compaction pressure on recycling AZ91 magnesium alloy formed via powder metallurgy. The AZ91 alloy was compacted at compaction pressures of 350 MPa, 400 MPa, and 450 MPa. The green density and sintering density of the alloy was increased with an increase in the compaction pressure. The optimum value for recycling AZ91 alloy was 450 MPa of compaction pressure. Burke et al. [78] analyzed the effects of compaction pressure on the mechanical characteristics of magnesium alloy (AZ31) formed via powder metallurgy at variable compaction pressure, i.e., 200 MPa, 300 MPa, 400 MPa, and 500 MPa. The theoretical density of the magnesium alloy was increased with compaction pressure. The hardness of the magnesium alloy was increased by increasing the compaction pressure. With the increase in compaction pressure, the samples are in a highly stressed state, which enables small dimensional changes to occur in the magnesium alloy sample—the influence of compaction pressure on the density of the composite (Figure 3f). However, Figure 3f shows conclusive evidence that the increase in sintering density defined the effectiveness of compaction pressure, which acts as a function of eliminating the micro-pores/voids, thereby reducing the porosity and ultimately increasing the mechanical properties.

However, the statistical data obtained after the analysis of Taguchi DOE are given below in Table 2. The outcomes revealed that the maximum value of the SN ratio for microhardness was obtained for the S8 sample, which showed that the optimal value of compaction pressure, combined with sintering temperature and sintering time, results in grain refinement and porosity reduction [77]. Moreover, sintering temperature and sintering time have a mixed effect on microhardness. Microhardness first increased with the increase in the sintering temperature and sintering time. However, with a further increase in sintering temperature and sintering time, microhardness is reduced because of recrystallization and grain growth in the material [77,78]. Therefore, the optimum set of process parameters that were used for the fabrication of the Mg-based composite is the third level of compaction pressure (450 MPa), the second level of sintering temperature (450°), and a sintering time of 90 min. The analysis of variance data for hardness is depicted in Table 3. The F and *p* values in the analysis of variance data are the measure of the effectiveness of the parameters. A parameter with high effectiveness has a high value of F and a low value of *p*. F-values also confirm the same order of the effectiveness of parameters, as obtained from response data for means for microhardness. The regression equation for hardness obtained after the ANOVA analysis is given below. The optimum value of hardness, which is obtained using the regression equation, with a compaction pressure of 450 MPa, sintering temperature of 450°, and sintering time of 90 min, is 81.41 Hv [77].

*H* = −37.84 − 2.342 *XX* + 1.775 *Y* + 2.548 *Z* + 0.002729 *XX*2 − 0.002919 *Y*2 − 0.002248 *Z*2 + 0.001971 *XXY* − 0.004873 *XXZ*,

Here, H = hardness (Hv), X = compaction pressure (MPa), Y = sintering temperature (°), and Z = sintering time (minutes). The experimental validation of the results obtained from regression equations for hardness was conducted. The outcomes revealed that the percentage error between the experimental results and results obtained from the regression equation is 4.3% for microhardness. Thus, regression equations provide satisfactory results and can be used to determine the value of hardness at other sets of parameters.

### 3.2. Types of Reinforcement

**Figure 4:**
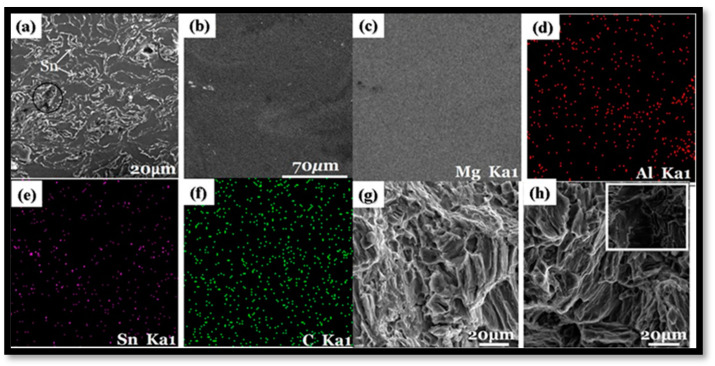
SEM micrograph of the composite (**a**) Mg-1wt.%Al-1wt.%Sn-0.18wt.%GNP, (**b**–**f**) X-ray mapping of Mg-1wt.%Al-1wt.%Sn-0.18wt.%GNP, and (**g**,**h**) fracture image of Mg-1wt.%Al-1wt.%Sn-0.18wt.%GNP [82].

**Figure 5:**
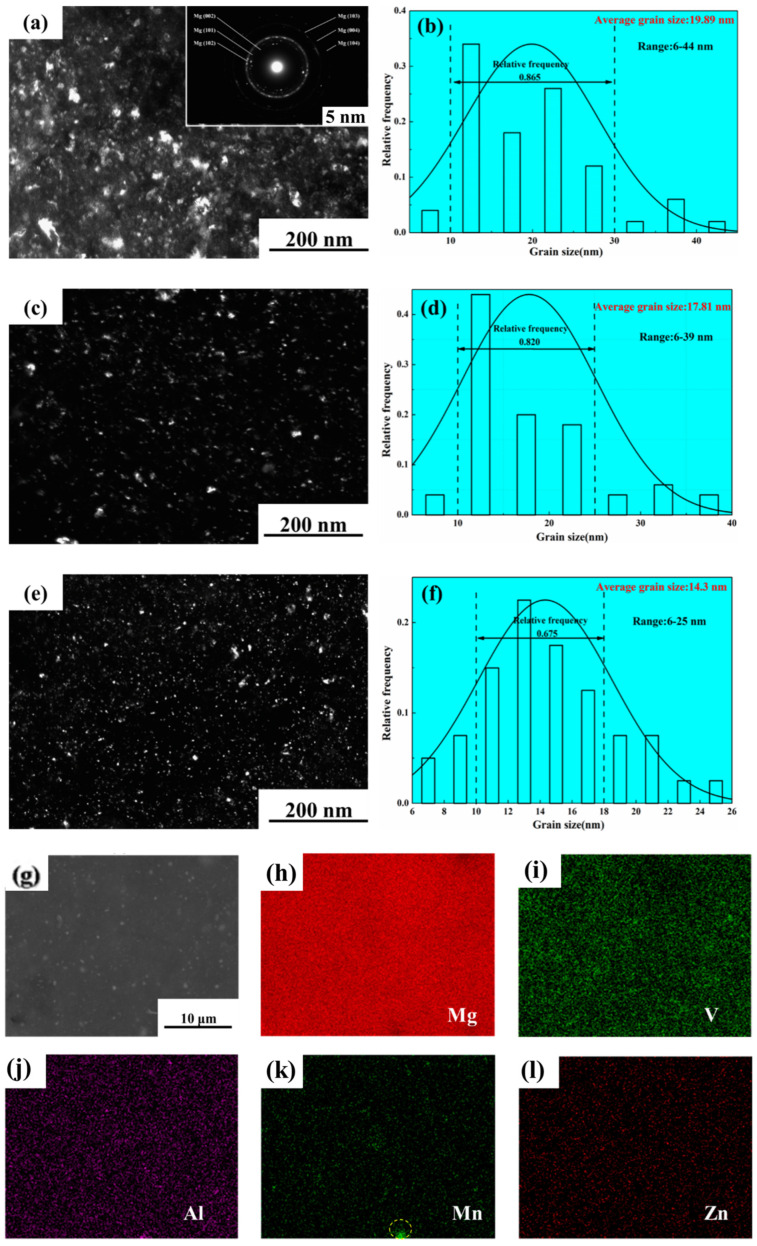
TEM image relating the grain distribution for (**a**,**b**) 5 wt.%, (**c**,**d**) 7.5 wt.%, and (**e**,**f**) 10 wt.% of vanadium. (**g**–**l**) SEM image and EDS mapping for 5 wt.% of vanadium in the AZ31 alloy, revealing the uniform dispersion of the reinforcing agent, along with the other elements, such as Al, Mn, and Zn [83].

The types of reinforcement are critical in predicting the behavior of composite materials. The characteristics of the reinforcing elements and matrix material have a substantial influence on the final characteristics of the composite. The types of reinforcement are also crucial in allowing the uniform dispersion of reinforcement in the matrix material throughout [79]. The uniform dispersion of reinforcement in the matrix material leads to improvement in the mechanical and corrosion characteristics of the composite by controlling porosity (%) [80]. Duarte et al. [81] analyzed the effects of niobium oxide (Nb_2_O_5_) reinforcement in the magnesium matrix on the mechanical characteristics formed via powder metallurgy. The magnesium was blended with 1 wt.%, 2 wt.%, and 4 wt.% of Nb_2_O_5_ in the ball mill. The microhardness of the green compact was increased with the increasing percentage of niobium oxide. However, after sintering, the microhardness of the composite was reduced with the addition of niobium oxide in the matrix material. Energy-dispersive spectroscopy (EDS) verified the homogenous dispersion of reinforcement in the matrix with slight oxidation. Rashad et al. [82] investigated the effects of reinforcement with graphene in the magnesium alloy (Mg–1%Al–1%Sn alloy) on the mechanical characteristics formed via powder metallurgy. The SEM image of Mg: 1 wt.%, Al: 1 wt.% Sn: 0.18 wt.%, and GNP depicted that Sn was evenly distributed throughout the matrix (Figure 4a). Due to oxidation during the sintering process, the black component exists. However, due to their low composition, Al and GNPs are difficult to identify using energy-dispersive X-ray spectroscopy (EDS). In order to establish the existence and distribution of Al and GNPs in the composite, X-ray mapping was performed in Figure 4b–f. It is evident in Figure 4f that GNPs are consistently distributed throughout the matrix and function as a strong reinforcing filler to stop the composite from deforming. The fractured morphology of the alloy exhibited ductile behavior, with many tearing ridges and dimples (Figure 4g). Some GNP particles are present, which are responsible for the low failure strain (Figure 4h). The 0.18 wt.% of graphene in magnesium alloy enhanced the mechanical characteristics of composites, i.e., 272 MPa and 213 MPa of ultimate tensile strength and 0.2% yield strength, respectively, which is higher than those of magnesium alloy. Sun et al. [83] analyzed the effects of reinforcement with vanadium (V) in the AZ31 alloy (magnesium alloy) on the mechanical characteristics formed via powder metallurgy. The magnesium alloy was blended with 5 wt.%, 7.5 wt.%, and 10 wt.% of vanadium in the ball mill. The theoretical density and porosity of the composite were enhanced with an increase in the weight fraction of vanadium. The TEM image relating the grain distribution for each weight fraction showcases the hindrance in grain growth with the addition of vanadium (Figure 5a–f). The SEM image, along with EDS mapping, revealed the presence of an intermetallic compound (Mn) and the uniform dispersion of vanadium in the Mg matrix (Figure 5g–l). The microhardness of the composite was improved with an increase in the amount of vanadium, with a maximum of 10 wt.% of vanadium, i.e., 106 Hv. Khanra et al. [84] analyzed the effects of hydroxyapatite (HAp) reinforcement in the magnesium matrix on the mechanical characteristics formed via powder metallurgy. The magnesium material is blended with 0 wt.%, 5 wt.%, 10 wt.%, and 15 wt.% of HAp in the ball mill. The XRD identified the formation of the composite’s MgO layer, enabling the interaction of matrix and reinforcing particles in the composite. The mechanical characteristics of the composite, i.e., UTM (146 MPa) and YS (137 MPa), were observed for 10 wt.% of HAp. Still, beyond that, there exists a reduction in yield strength with HAp. Further, the microhardness was also enhanced with an increase in the amount of HAp.

**Figure 6:**
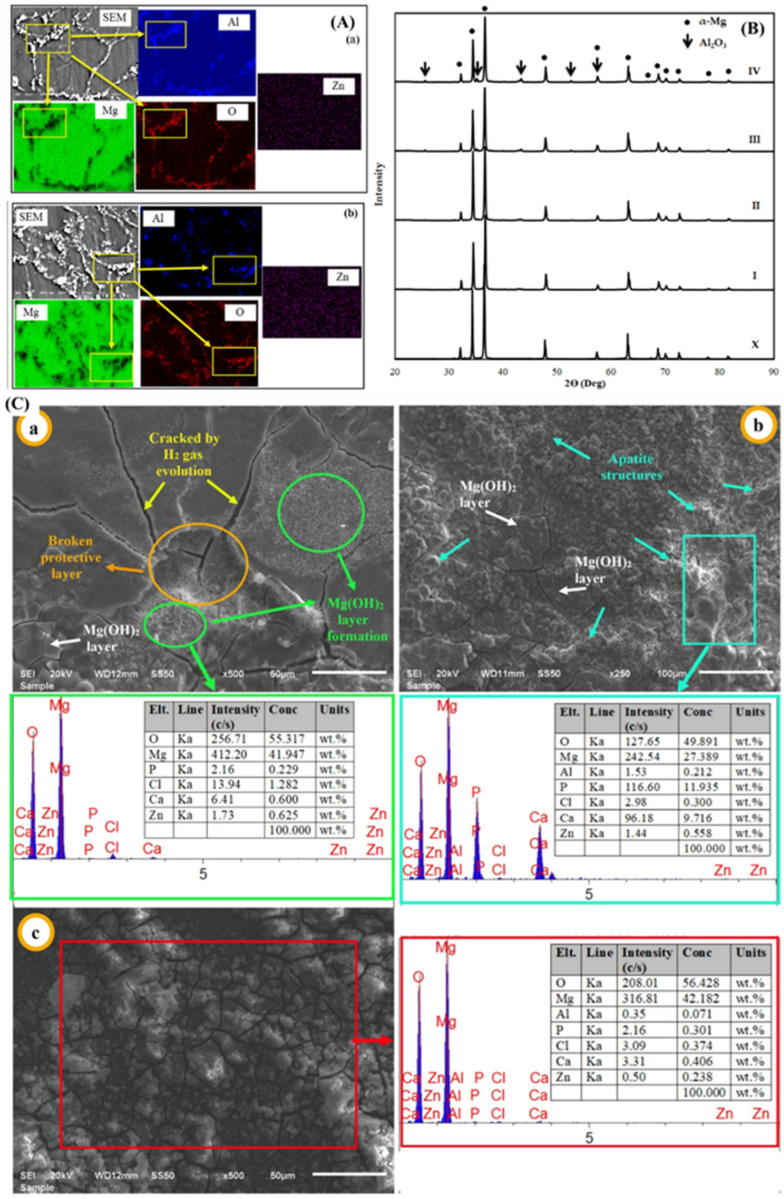
(**A**) FESEM image and EDS mapping for (**a**) 2 wt.% of Al_2_O_3_ and (**b**) 8 wt.% Al_2_O_3_ of Mg composites. (**B**) XRD analysis of the composite depicting (X = 0 wt.%, I = 2 wt.%, II = 4 wt.%, III = 6 wt.%, IV = 8 wt.%). (**C**(**a**–**c**)) shows the EDS analysis of the samples after immersion, showcasing the different elemental components in the composite and showing that pitting corrosion is found in the presence of a high Al_2_O_3_ content [100].

In MMCs, reinforcement involves the majority of the applied load, with the matrix binding the reinforcements together and providing the load distribution over the individual reinforcement particles [84,85,86,87,88]. The powder metallurgy technique is widely regarded as a low-cost approach to fabricating MMCs that is easy and highly suitable for large-scale production [89,90]. The fabrication of MMCs via powder metallurgy involves no chemical reaction between matrices and reinforcement with low porosity and obtains a uniform composition of the reinforcing particles [91,92,93,94,95]. The strong bond between the particle reinforcements and the metal matrix allows the load to be transferred and distributed from the matrix to the reinforcement without failure [96,97,98]. Further, the addition of ceramic particles improves the mechanical and frictional behavior of the composite [99]. The reinforcing materials are silicon carbide, alumina, aluminum oxide, and boron carbide. Research studies have confirmed that different reinforcements impact Mg properties differently. Ercetin et al. [100] analyzed the effects of reinforcement with Al_2_O_3_ in the Mg_2_Zn alloy on the mechanical characteristics and microstructure formed via powder metallurgy. The magnesium alloy was blended with 0 wt.%, 2 wt.%, 4 wt.%, 6 wt.%, and 8 wt.% of Al_2_O_3_. The theoretical density of the composite was enhanced with Al_2_O_3_. The SEM image depicted the uniform composition of reinforcement in the matrix, and no pores were observed in the microstructure of all samples (Figure 6A(a,b)). The grain size was reduced with the addition of Al_2_O_3_ to the composite, leading to an increase in the tensile strength and hardness of the composite. The XRD image showed that the Mg peak was seen initially, but increasing the amount of reinforcement led to the formation of an Al_2_O_3_ peak in the composite (Figure 6B). Figure 6C(a–c) shows the EDS analysis of the samples after immersion, showcasing the different elemental components in the composite and showing that pitting corrosion is found in the presence of a high Al_2_O_3_ content. The reduction in the elongation and corrosion resistance of the composite was observed with Al_2_O_3_. Sankar et al. [101] analyzed the effects of reinforcement with B_4_C in the AZ91 alloy (magnesium alloy) on the tribological performance formed using powder metallurgy. The magnesium alloy was blended with 5 wt.%, 10 wt.%, 15 wt.%, and 20 wt.% of B_4_C in a ball mill. The density and microhardness of the composite were improved with B_4_C. The wear resistance of the composite was improved with the increase in the amount of B_4_C.

**Figure 7:**
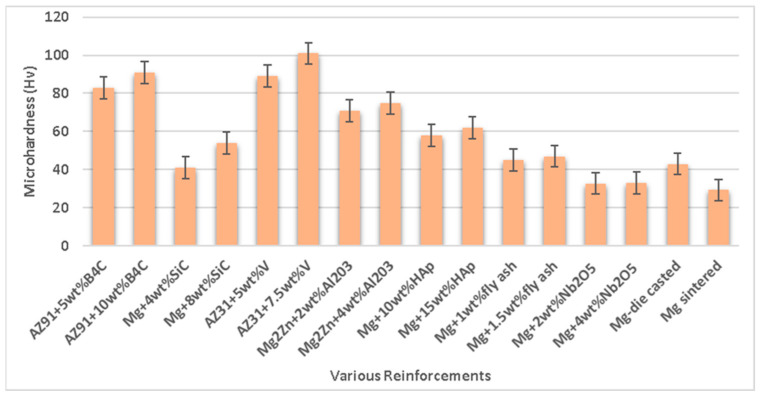
Variation in microhardness of composites subject to reinforcement.

The Orowan mechanism describes the effect of reinforcing particles on the strength of composite materials [102,103,104]. The evenly distributed ceramic particles (SiC, B_4_C, alumina) hinder dislocation movement, which acts as a function of improved strength [105]. However, superior adhesion and a distinct surface interface delay the detachment of reinforcement from the matrix material, which enhances the overall characteristics of the composites. Kumar et al. [106] investigated the effects of reinforcement with silicon carbide (SiC) in a magnesium matrix on the mechanical characteristics formed via powder metallurgy. The magnesium matrix was blended with 0 wt.%, 4 wt.%, 8 wt.%, and 12 wt.% of SiC in the ball mill. The density of the composite was observed to increase with the addition of silicon carbide due to the reduction in porosity and was found to be at its maximum, i.e., 1.79 g/cm^3^ for 12 wt.% of SiC. The compressive strength, Vickers hardness, and impact strength of the composite were increased with an increase in the amount of silicon carbide and maximum, i.e., 540 MPa, 71 Hv, and 5 J for 12 wt.% of SiC. Chand et al. [107] analyzed the effects of fly ash in the magnesium matrix material on the mechanical characteristics formed via powder metallurgy. The magnesium matrix was blended with 0.5 wt.%, 1 wt.%, 1.5 wt.%, and 2 wt.% of fly ash in a ball mill. The mechanical characteristics of the composite, such as ultimate tensile strength and yield strength, were increased with fly ash due to the reduction in porosity. But Young’s modulus, microhardness, and elongation (%) were increased up to 1.5 wt.% of fly ash in the matrix; however, beyond that limit, there exists a reduction in Young’s modulus, microhardness, and elongation (%) of the composite due to an increase in porosity. The influence of the types of reinforcement on the mechanical characteristics of Mg-based material composite is depicted in Figure 7.

### 3.3. Weight Fraction of Reinforcement

**Figure 8:**
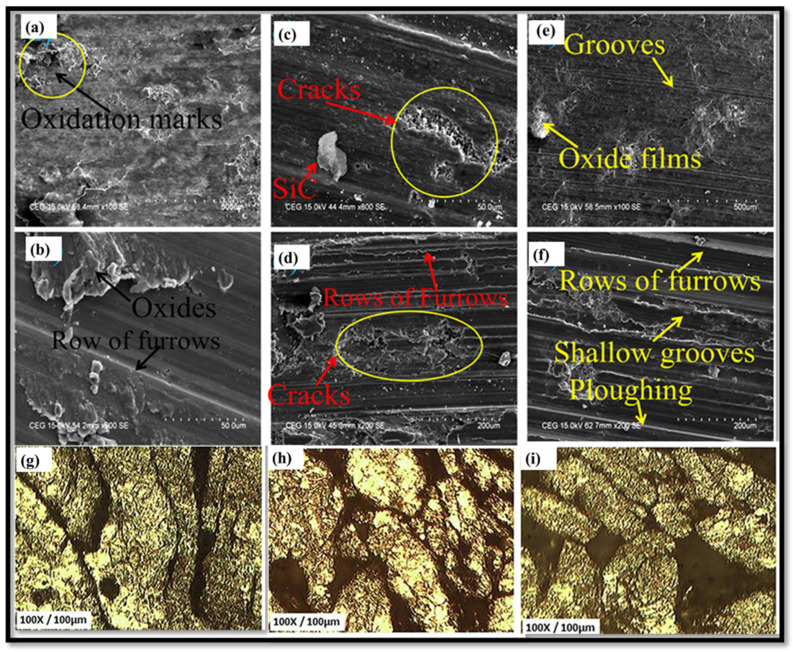
SEM micrograph of worn-out surfaces of Mg /SiC (**a**) Mg/SiC for 0.4 m/s at 5 N, (**b**) Mg/SiC for 0.4 m/s at 10 N, (**c**) Mg/SiC for 0.6 m/s at 5 N, (**d**) Mg/SiC for 0.6 m/s at 10 N, (**e**) Mg/SiC for 0.8 m/s at 5 N, and (**f**) Mg/SiC for 0.8 m/s at 10 N [109]. (**g**–**i**) Optical characterization of TiC-reinforced (3 wt.%, 6 wt.%, and 9 wt.%) magnesium composites [111].

The optimal volume or weight fraction value predicts the suitable mechanical characteristics for the desired applications. However, decreasing or increasing the volume or weight percentage of the reinforcement entails enhancing and degrading mechanical characteristics [108]. The amount of reinforcement, therefore, becomes critical in assessing the requisite mechanical characteristics of the composite and controlling the porosity (%). Kaviyarasan et al. [109] analyzed the wear behavior of magnesium reinforced with ceramics (SiC) (0.5 wt.%, 1 wt.%, 1.5 wt.%, and 2 wt.%) formed via powder metallurgy. The magnesium was blended with silicon carbide in a ball mill to create a uniform mixing of the reinforcement and matrix in the composite. The wear rate of the composite was analyzed at a variable sliding velocity (0.4 m/s, 0.6 m/s, and 0.8 m/s), corresponding to load variations of 5 N and 10 N. An SEM image of wear-out samples illustrated the reduction in wear rate with the addition of silicon carbide (Figure 8a–f). Along with an improvement in wear resistance, the hardness of the composite was also improved with an increase in the amount of silicon carbide. Kanthasamy et al. [110] analyzed the corrosion and mechanical characteristics of the magnesium alloy (AZ31) reinforced with groundnut shell ash particles (GSAp) (1 wt.%, 2 wt.%, and 3 wt.%) formed by powder metallurgy. The microhardness of the composite was enhanced up to 2 wt.% of GSAp (55.62 Hv) in the matrix. Beyond that, there is a reduction in hardness with the particles of groundnut shell ash. The micrograph depicted that the homogeneous dispersion of reinforcing material in the matrix was observed up to 2 wt.% of GSAp. Beyond that, the agglomeration of particles was observed, which caused cracks to appear on the surface, leading to a reduction in the values of the mechanical characteristics of the composite. The compressive strength was also reduced after 2 wt.% of GSAp in the matrix. The penetration rate of the composite was positively increased with the addition of groundnut shell ash particles and was higher than that of the AZ31 alloy. As a result, the corrosion rate of the composite was increased with the addition of groundnut shell ash particles. Annbuchezhiyan et al. [111] analyzed the mechanical characteristics and microstructure of magnesium alloy (AZ91D) reinforced with TiC (3 wt.%, 6 wt.%, and 9 wt.%) formed via powder metallurgy. Optical microscopy (OM) showed a fine lamellar grain structure that improved the mechanical characteristics of the composite (Figure 8g–i). Other than that, the homogenous dispersion of titanium carbide was observed in the matrix material in the composite. The microhardness and the corrosion resistance of the composite were found to be increased with titanium carbide in the matrix, as the titanium and carbide phases present in the composite hindered the plastic flow and dislocation in the matrix.

Reinforcements play a crucial role in determining the corrosion and wear resistance of composites. Magnesium is widely used in various industries due to its low density, high specific strength, and excellent machinability. However, its susceptibility to corrosion in various environments poses a significant limitation. Reinforcements, which are added to improve mechanical properties such as strength and wear resistance, can also influence the corrosion behavior of magnesium-based composites [112,113,114,115,116]. The effect of reinforcements on corrosion resistance is complex and depends on factors such as the type of reinforcement, its distribution, and interfacial bonding [117]. Ceramic particles, such as SiC, Al_2_O_3_, B_4_C, and TiC, are commonly used to reinforce Mg material that is chemically stable and improve the hardness and wear resistance of the composite. However, their impact on corrosion resistance can be dual [118]. SiC particles can act as galvanic sites when embedded in the Mg matrix, leading to localized corrosion around the reinforcement particles. This galvanic effect occurs because SiC creates a potential difference that accelerates the corrosion of the Mg-based materials [119,120,121,122,123]. However, Al_2_O_3_ reinforcements reduce the corrosion rate by forming a protective barrier and improving the uniformity of the passive film on the magnesium surface [124,125,126]. However, the high amount of reinforcement leads to cluster formation, ultimately introducing defects and exacerbating corrosion in the matrix material [127]. The presence of Al_2_O_3_ fibers in the AM60 magnesium alloy leads to increased corrosion rates, whereas increasing the amount of Al_2_O_3_ leads to an improvement in corrosion resistance [127]. Further, the addition of B_4_C in Mg imparts high hardness and thermal stability, which leads to an enhancement in the wear resistance of composites by reducing material loss during sliding or abrasive wear [128]. Moreover, the ceramic particles effectively improve wear resistance by acting as load-bearing elements, thereby reducing direct contact with abrasive surfaces.

Metallic reinforcements, such as stainless steel, Ti fibers, and aluminum particles, can significantly alter the corrosion and wear behavior of composites, as these reinforcements improve mechanical properties by acting as cathodic sites and accelerating galvanic corrosion [129]. Furthermore, Ti and stainless steel fibers enhance wear resistance by improving the load-bearing capacity of the composite. Magnesium composites reinforced with Ti particles exhibit superior wear resistance under dry sliding conditions compared to unreinforced magnesium alloys [130]. However, stainless steel fibers provide excellent wear resistance by improving the toughness and reducing the material loss during wear [131]. The presence of metallic reinforcements can increase the density of the composite, which may limit their use in weight-sensitive applications. Furthermore, carbon-based materials, such as CNT, graphene, and carbon fiber, are increasingly being used to reinforce magnesium composites, generally improving the wear resistance and mechanical properties of MMCs; however, their effect on corrosion resistance is variable [132] since graphene acts as a cathodic to Mg, promoting galvanic corrosion if not uniformly distributed [133]. However, functionalized CNT enhances corrosion resistance by improving the dispersion of reinforcements and the quality of the passive film on the magnesium matrix [134].

Furthermore, the interfacial bonding between the reinforcement and matrix is critical in determining the corrosion and wear resistance of the composite. Weak bonding or the presence of voids and cracks at the interface can serve as pathways for corrosive agents, accelerating localized corrosion [135]. However, surface treatments of reinforcements, such as coating with silane or metallic layers, can enhance interfacial bonding and reduce corrosion rates [136]. Coating SiC particles with nickel or aluminum before incorporating them into the Mg matrix can mitigate galvanic corrosion by reducing the potential difference between the reinforcement and the matrix [137]. However, the size and distribution of reinforcement particles play a significant role in the corrosion and wear behavior of composites [18]. Uniformly distributed fine particles tend to enhance corrosion resistance by improving the homogeneity of the microstructure and the passive film. In contrast, coarse or clustered particles can create stress concentration points and galvanic cells, leading to localized corrosion [138]. However, Mg reinforced with nano-Al_2_O_3_ particles demonstrated superior corrosion resistance compared to Mg reinforced with micron-sized particles due to the uniform dispersion and better interfacial bonding of the nano-reinforcements [139].

**Figure 9:**
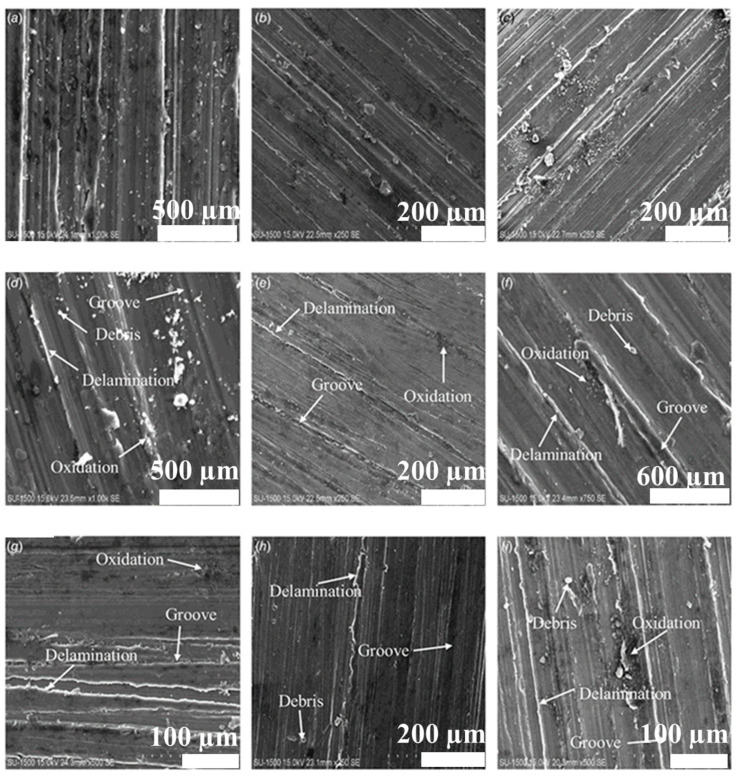
Surface morphology of worn-out sample of Mg/0.5 wt.% BN composites at a normal load of 5 N and a sliding velocity of (**a**) 0.6 m/s, (**d**) 0.9 m/s, and (**g**) 1.2 m/s; composites at a normal load of 7 N and a sliding velocity of (**b**) 0.6 m/s, (**e**) 0.9 m/s, and (**h**) 1.2 m/s; and composites at a normal load of 10 N and a sliding velocity of (**c**) 0.6 m/s, (**f**) 0.9 m/s, (**i**), 1.2 m/s [143].

Jiang et al. [140] investigated the wear performance of Mg reinforced with B_4_C (10 wt.%–20 wt.%) prepared using a powder metallurgy approach and showed an improvement in wear resistance, i.e., 5.7039 for 35 N at 20 wt.% of B_4_C [140]. Kaviti et al. [141] investigated the wear performance of an AZ31/alumina composite prepared via powder metallurgy by varying sliding speed (0.6 m/s, 0.9 m/s, and 1.2 m/s), normal load (5 N, 7 N, and 10 N), and sliding distance (500 m, 1000 m, and 1600 m). However, as the load increased, the friction coefficient decreased, whereas sliding velocity and distance had little effect. Jayaraman et al. [142] examined the tribological behavior of AZ31/CNTs (0.33 wt.%, 0.66 wt.%, and 1 wt.%) prepared via powder metallurgy by varying the normal load (15.7 N, 25.5 N, 35.32 N) while keeping the sliding velocity constant (1.04 m/s). The outcomes show that CNT up to 0.66 wt.% improved the wear resistance of the composite. Kaviti et al. [143] examined how boron nitride (BN) (0 wt.%–2.5 wt.%) affected the wear behavior of Mg composite prepared via powder metallurgy. The outcomes revealed that Mg reinforced with 0.5 wt.% of BN has a lower wear rate and friction than Mg/1.5 wt.% BN and 2.5 wt.% BN composites. SEM images of a worn Mg/0.5 wt.% BN composite at different sliding speeds and normal loads are shown in Figure 9.

**Figure 10:**
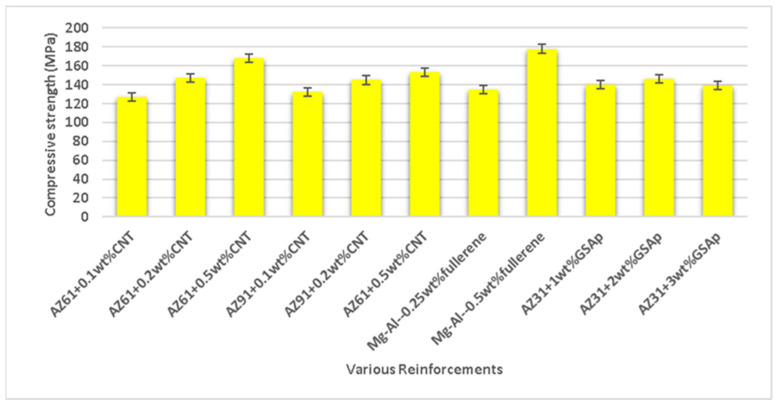
Compressive strength of composites with reinforcement.

Turan et al. [144] analyzed the mechanical and corrosion characteristics of a magnesium (Mg-1wt.%Al) alloy reinforced with fullerene (0.50 wt.%) formed via powder metallurgy. Bragg’s diffraction peak (resembling hexagonal Mg phase) is around 15^o^ to 75^o^, confirming the existence of fullerene in a Mg alloy. Field emission scanning electron microscopy (FESEM) shows no macrostructural defects appearing on the surface of the magnesium alloy reinforced with fullerene. The homogeneous dispersion of fullerene in the magnesium alloy matrix is present without the appearance of defects or cracks with fullerene. The mechanical behavior of the composite, i.e., Vickers hardness (56 Hv), ultimate compressive strength (296 MPa), and compressive failure strain (15.72%), appeared to be enhanced with fullerene. The wear rate of the composite was assessed across sliding velocity (48 mm/s and 96 mm/s) and applied load (5 N, 10 N, and 20 N) values. The wear resistance was increased with the addition of fullerene to the composite. The corrosion resistance of the composite was enhanced with fullerene in the magnesium alloy. Say et al. [145] analyzed the mechanical and corrosion characteristics of the magnesium alloy (AZ91 and AZ61) reinforced with carbon nanotubes (CNTs) (0.1 wt.%, 0.2 wt.%, and 0.5 wt.%) formed via powder metallurgy. XRD confirmed the presence of the β-Mg_17_Al_12_ phase in the magnesium alloy. The compressive strength and yield strength increased with carbon nanotubes for the AZ31 alloy composite (Figure 10). The yield strength of the AZ91 alloy composite was decreased, whereas the compressive strength was increased with the addition of carbon nanotubes to the composite. A continuous increase in the porosity and reduction in ductility was observed for magnesium alloy composites (AZ61 and AZ91) with the addition of carbon nanotubes.

### 3.4. Volume Fraction of Reinforcement

**Figure 11:**
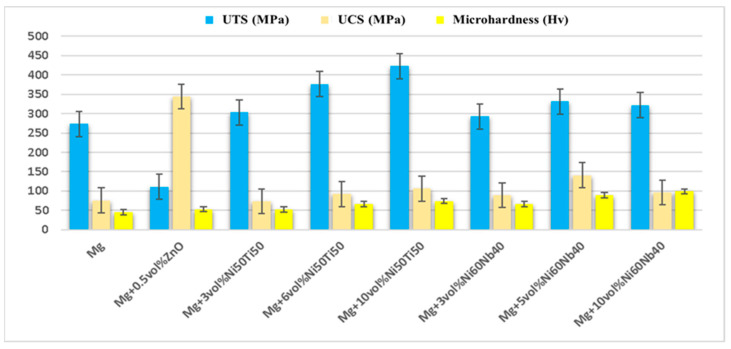
Mechanical properties of Mg-based material composites formed using different reinforcements (vol.%) in MMCs.

Like the weight fraction, the volume fraction is crucial in analyzing the mechanical characteristics of the Mg-based composite. The studies demonstrate the improvement in the mechanical characteristics of Mg-MMCs via volume fraction of reinforcement. Kondoh et al. [146] analyzed the effects of volume fractions of reinforcement (0.95–1.43 vol.% of CNTs) on the mechanical characteristics and microstructure of magnesium formed via powder metallurgy. The microstructure analysis via TEM showed the existence of a thin layer of MgO between the unbundled nanoparticles and the α-phase, creating the strong interaction between the nanoparticles that led to effective transfer in tensile loading. The uniform dispersion of reinforcement material in the matrix was analyzed using SEM. The tensile strength and yield strength of the composite were observed to increase with an increase in the number of carbon nanotubes. Further, the elongation (%) was reduced with the amount of carbon nanotubes due to MgO layer formation, which hinders the increase in ductility. Selvam et al. [147] analyzed the effect of 0.5 vol.% of zinc oxide on the mechanical and wear behavior of an Mg alloy prepared via PM. The microhardness and ultimate tensile strength (UTS) of the composite were increased with the addition of 0.5 vol.% of ZnO to the magnesium matrix composite. However, the 0.2% yield strength was reduced with the addition of zinc oxide in the matrix material. The ultimate tensile and compressive strength of the composite was enhanced with the addition of reinforcement in the composite. The wear rate of the composite was analyzed, corresponding to sliding velocity (0.6 m/s, 0.9 m/s, and 1.2 m/s) and normal load (2 N, 7.5 N, and 10 N). The wear rate for sliding velocities (0.6 m/s and 0.9 m/s) was increased due to the formation of wear debris, scratches, and groves; however, there was a reduction in the wear rate beyond that due to the formation of the oxides. Sankaranarayanan et al. [148] analyzed the influence of the volume fraction of metallic glass (Ni_50_Ti_50_) on the mechanical characteristics of Mg composite formed via powder metallurgy (3 vol.%, 6 vol.%, and 10 vol.%). Optical microscopy suggested that a reduction in the grain size of the composite was observed with the addition of metallic glass to the matrix material. The mechanical characteristics of the composite, i.e., the ultimate tensile strength and 0.2% yield strength, were found to be higher with an increase in the amount of metallic glass. However, the failure strain of the composite appeared to be decreased with the increase in the amount of metallic glass. The microhardness, ultimate tensile strength, and compressive strength of the composite increased with the increase in the amount of reinforcing agent in the matrix material. Jayalakshmi et al. [149] analyzed the effects of glass particles (Ni_60_Nb_60_) on the mechanical characteristics of magnesium formed via powder metallurgy (3 vol.%, 5 vol.%, and 10 vol.%). Optical microscopy suggested that a reduction in the grain size of the composite was observed up to 5 vol.% of (Ni_60_Nb_60_); however, beyond that, a slight increase in grain size occurred. The mechanical characteristics of the composite, i.e., ultimate tensile strength and 0.2% yield strength, were found to be higher with an increase in the amount of metallic glass. However, the failure strain of the composite appeared to be reduced with the reduction in metallic glass. The UTS, microhardness, and ultimate compressive strength (UCS) of the composite were increased with an increase in the amount of reinforcement in the matrix material (Figure 11). The failure strain of the composite was observed to be lower compared to that of the magnesium matrix by increasing the percentage of reinforcement in the magnesium matrix. The XRD image showed that no reaction occurred between the magnesium matrix and the glass particles.

### 3.5. Particle Size of Matrix and Reinforcement

**Figure 12:**
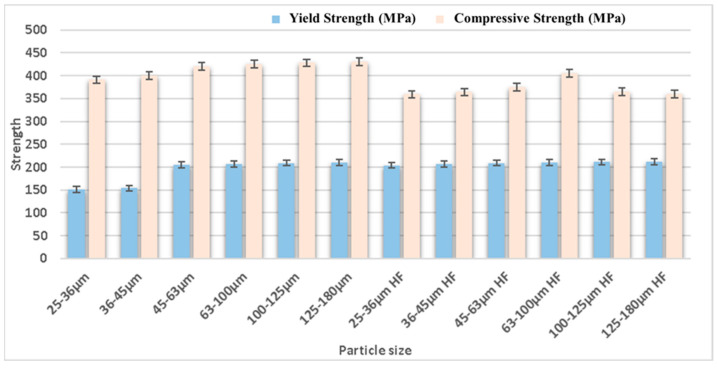
Variation in compressive strength of composites with different particle sizes of reinforcement in MMCs.

The particle size of the reinforcement and matrix affects the surface morphology and mechanical behavior of Mg-based material formed using the powder metallurgy approach. The strength and porosity of the composite material are directly related to the reinforcement and matrix particle size [150,151,152,153,154,155]. Further, the size of the void developed in the composite material influences the strength and porosity of the composite material, which is critical to examine [156,157]. Dvorsky et al. [158] analyzed the effects of particle size on the corrosion resistance and mechanical characteristics of WE43 (Mg-4Y-3REE-Zr) alloy and WE43 (Mg-4Y-3REE-Zr) reinforced with HF composite. The mechanical characteristics of the alloy and composite components were varied, along with the particle size, i.e., 25–36 µm, 36–45 µm, 45–63 µm, 63–100 µm, 100–125 µm, and 125–180 µm. The yield strength of the alloy and composite was enhanced with an increase in the particle size; however, the ultimate compressive strength of the alloy increased up to 100–125 µm and then decreased with an increase in particle size up to 125–180 µm. The ultimate compressive strength of the composite was increased initially up to 63–100 µm but then decreased with an increase in particle size up to 125–180 µm. The ultimate tensile strength of the alloy increased with the increase in particle size. Still, the ultimate strength of the composite increased up to 63–100 µm, and then there was a sudden reduction in ultimate tensile strength with an increase in particle size of up to 125–180 µm. The ductility (%) of the alloy and composite was enhanced with the particle size. The corrosion resistance of the alloy and composite was reduced with the increase in particle size. The ignition temperature of the alloy was decreased with an increase in the size of the particles. Rahmani et al. [159] analyzed the effects of the matrix particle size on the mechanical characteristics of a magnesium alloy (AZ91) reinforced with B_4_C (5 wt.%, 10 wt.%, 15 wt.%, and 20 wt.%) formed via powder metallurgy. The particle size of the matrix was taken as 10 µm and 60 µm to form an AZ91/B_4_C composite. The porosity of AZ91 with a particle size of 60 µm reinforced with different percentages of B_4_C was observed to be lower than that of AZ91 with a particle size of 10 µm reinforced with varying percentages of B_4_C. Therefore, the density and microhardness of the composite appeared to be higher for the matrix material with a particle size of 60 µm than the matrix material with a particle size of 10 µm. The influence of particle size of reinforcement on the mechanical properties of the composite is shown in Figure 12.

### 3.6. Sintering Temperature

The sintering temperature is an important process parameter that strengthens the green compact. The sintering temperature and time are directly related to the diffusion of reinforcing and matrix material in the composites that enhance the strength of the final product obtained via powder metallurgy [160,161,162]. Therefore, the increase in grain growth and diffusion bonding depends upon the strength of the green compact, which is related to the sintering temperature. On increasing the sintering temperature, the diffusion bonding improves between the reinforcing and matrix material, while the grain growth that occurs reduces the strength of the material [163,164,165]. Therefore, the selection of sintering temperature is critical in powder metallurgy. Zhu et al. [166] analyzed the effect of the sintering temperature on the mechanical characteristics and surface topography of an Mg alloy (AZ91) by varying the sintering temperatures, i.e., 350 °C, 400 °C, and 450 ºC. The microstructure showed that the grain size of the AZ91 alloy increased with the increase in the sintering temperature, whereas β-phase (Mg_17_Al_12_) precipitation content and GND density were observed to decrease. The compressive strength of the alloy was reduced with the increase in the sintering temperature, whereas the fracture strain and ultimate compressive strength (UCS) of the alloy were increased, which lowered the content of β-Mg_17_Al_12_ and MgO and coarse grains with the increase in sintering temperature. Ma et al. [167] analyzed the effects of the sintering temperature on the mechanical characteristics of the ZK60 magnesium alloy by varying the sintering temperature, i.e., 450 °C, 500 °C, 550 °C, and 600 ºC. The microstructure demonstrated the presence of equiaxed grains and the α-Mg phase in all samples of the alloy with varying sintering temperatures.

The sintering temperature (450 °C and 500 ºC) enabled the uniform dispersion of material powder that increased the hardness and yield strength of the alloy. Still, beyond that limit, a reduction in hardness and yield strength exists due to the decrease in the zinc element in the melt. The compressive strength of the alloy was increased up to 550 °C, but beyond that temperature, a reduction in the compressive strength occurred. The failure strain was linearly varied with the increase in sintering temperature and observed to a maximum of 600 ºC. Durai et al. [168] investigated the effects of the sintering temperature on the mechanical characteristics of a magnesium–zirconium (Mg-Zr) alloy by varying the sintering temperature, i.e., 450 °C, 500 °C, and 550 ºC. A reduction in the porosity (%) of the alloy was observed by increasing the sintering temperature. The hardness and tensile strength of the alloy were improved by increasing the sintering temperature due to a reduction in porosity. The density of the alloy was increased up to 500 °C, but beyond that temperature, a reduction in density was observed by increasing the sintering temperature. The magnesium alloy’s ductile cleavage fracture (combination of brittle and plastic deformation) was observed with an increasing sintering temperature. Kumar et al. [77] analyzed the effect of sintering temperature when recycling AZ91 magnesium alloy formed via powder metallurgy at varying sintering temperatures, i.e., 673 °C, 723 °C, and 773 °C. The theoretical density of the composite was observed to be increased up to 723 °C, but beyond that temperature, there occurred a reduction in density with the increase in sintering temperature. The optimum sintering temperature at which to recycle AZ91 magnesium alloy is 723 °C.

**Figure 13:**
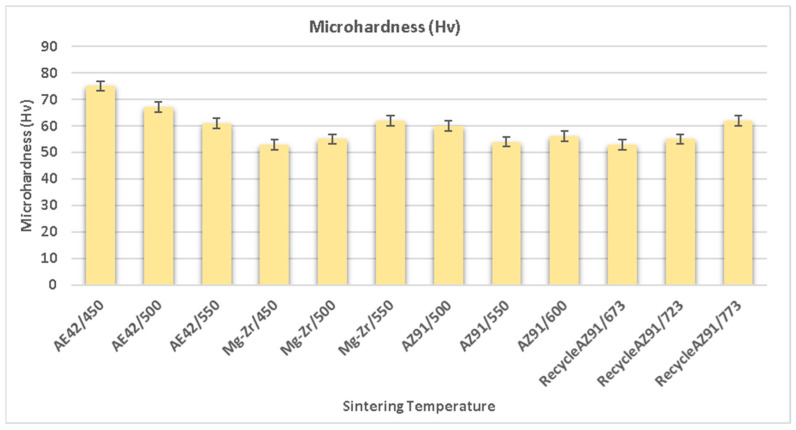
Variation in microhardness of composites at different sintering temperatures in MMCs.

In the sintering process, the diffusion of the material occurs in pores that enable the chemical and physical bond at the surface interface of reinforcing and matrix powder material, which improves the strength of the composite material. The research suggests that the diffusion of material varies with temperature and time and illustrates that the sintering time and sintering temperature determine the diffusion and expansion rate of powder materials in the composite [169,170,171,172,173,174,175]. The literature shows that a suitable sintering time also enhances the strength of the composite. Zhou et al. [176] analyzed the effects of the sintering temperature on the mechanical characteristics of the AZ91 alloy by varying the sintering temperature, i.e., 450 °C, 500 °C, 550 °C, and 600 ºC. The hardness, tensile strength, and density are observed to increase up to 550 ºC, but beyond that temperature, there exists a reduction in mechanical characteristics due to an increase in porosity that generates tiny holes in the alloy sample. The microstructure depicted that the β-Mg_17_Al_12_ phase was distributed around the grain boundaries that enhance the resistance to corrosion characteristics of the alloy. The β-Mg_17_Al_12_ and α-Mg phases were obtained in white and gray matter distributed along the grain boundary. Minarik et al. [177] analyzed the effects of the sintering temperature on the mechanical characteristics of the AE42 magnesium alloy by varying sintering temperatures, i.e., 400 °C, 450 °C, 500 °C, and 550 °C. The microhardness of the alloy was observed to decrease with the increase in the sintering temperature due to grain growth. The increase in the sintering temperature was shown to result in the loss of mechanical characteristics of the alloy due to an increase in grain size. Annur et al. [178] analyzed the effects of the sintering temperature (580 °C and 630 °C) on the mechanical characteristics of an Mg-Zn-Ca alloy and an Mg-Zn-Ca/carbamide composite prepared via PM. The XRD image showed the MgO phase in all samples of the Mg-Zn-Ca alloy at all sintering temperatures, and the CaO phase was obtained in all the samples of the Mg-Zn-Ca/carbamide composite at all sintering temperatures. The compressive strength of the composite decreased at sintering temperatures beyond 580 °C in all samples due to an increase in porosity. SEM confirmed that high sintering temperatures resulted in higher porosity, which reduced the mechanical characteristics. Burke et al. [78] analyzed the effects of the sintering temperature on the mechanical characteristics of a magnesium alloy (AZ31) formed via powder metallurgy at varying sintering temperatures, i.e., 500 °C, 550 °C, and 600 °C. The small dimensional changes occurred in the sample subject with an increase in sintering temperature due to an increase in densification. The theoretical density and Rockwell hardness of the magnesium alloy were reduced with an increase in the sintering temperature (Figure 13).

The interface between the Mg as a matrix and the reinforcement plays a pivotal role in determining the mechanical properties of Mg-based materials. The strengthening mechanisms at the interface are governed by several interrelated factors, including the formation of the interfacial layer, the interfacial binding force, and the stability of the interface during sintering [179]. Each of these aspects contributes uniquely to the overall performance of the composite.

*1. Formation of the Interfacial Layer:* During the sintering process, a reaction layer developed at the boundary interface between the reinforcement and matrix [180]. However, the characteristics of this layer, including its thickness, composition, and compatibility with both the matrix and reinforcement, can either improve or detract from the mechanical properties of the composite [181]. Moreover, an optimally designed interfacial layer enhances the efficiency of load transfer by facilitating a smooth transition in mechanical properties between the matrix and the reinforcement [182]. Conversely, if the interfacial layer is too thick or brittle, it may become a stress concentration at the interface, resulting in early failure [181,182]. The interfacial layers serve as obstructions to dislocation movement, thus increasing the strength of the composite [183]. However, the presence of nano-MgO particles at the interface was identified in the Mg composite reinforced with reduced graphene oxide. These particles increase the interfacial bonding strength between reduced graphene oxide and the matrix, leading to simultaneous improvements in strength and ductility [184].

*2*. *Interfacial Binding Force:* The efficiency of stress transfer from the matrix to the reinforcement is dictated by the strength of the interfacial bond [185]. A composite’s load-bearing capacity is improved through robust chemical bonding or mechanical interlocking at the interface. However, to attain an ideal binding force during sintering, a careful balance of reactivity must be maintained; too many reactions can compromise the interface’s integrity, while inadequate bonding results in diminished load transfer and lower composite strength [186]. Therefore, the effective load transfer and overall performance of the composite hinge on the strength of the bonding force between the matrix and reinforcement. Strong interfacial bonding guarantees that stress is effectively conveyed from the softer matrix to the stiffer reinforcement, thus boosting the composite’s strength and stiffness [187]. Reinforcing Mg-based materials with few layers of MXene results in strong interfacial bonding, which contributes to significant improvements in mechanical properties [188]. Additionally, applying surface coatings, i.e., nickel, copper, or ceramic layers, in the reinforcement phase can improve the wettability and chemical compatibility between the reinforcement and the magnesium matrix, resulting in stronger interfacial bonding and improved mechanical properties [189].

*3. Interfacial Stability*: Maintaining the integrity of the composite during sintering heavily relies on the thermal and chemical stability at the interface of the composite. If reactions between the reinforcement and matrix are not controlled, then it will result in unwanted voids or phases that compromise the mechanical properties of the composite [190]. By optimizing sintering parameters, such as temperature and time, interfacial stability can be maintained, thereby ensuring reliable performance [191]. Therefore, it is crucial to uphold interfacial stability during sintering to avoid the creation of brittle phases, thereby safeguarding the composite’s integrity. Elevated sintering temperatures may trigger interfacial reactions that yield brittle intermetallic compounds, negatively impacting the mechanical characteristics of composites [192]. However, the Mg-Ti composites produced via powder metallurgy at lower sintering temperatures sustained interfacial stability and averted the development of brittle phases, thus improving the mechanical performance of the composite [193].

Further, the sintering temperature influences the various parameters, i.e., particle size, grain refinement, and reinforcement distribution, that critically affect the mechanical behavior of the Mg-based material [194]. These factors are as follows:

*1. Grain Growth and Microstructure Refinement*: The growth of grains in Mg-based materials is affected by the sintering temperature. Increasing sintering temperatures can enhance the mobility of grain boundaries, which may cause the microstructure to coarsen [195]. However, the use of smaller particle sizes can mitigate excessive grain growth by offering additional nucleation sites, leading to finer microstructures [196]. As the presence of smaller reinforcement particles like nano-SiC or nano-Al_2_O_3_ often creates a pinning effect at grain boundaries, limiting their movement and ensuring a refined microstructure is preserved, even at higher sintering temperatures [196].

*2. Reinforcement Distribution and Bonding*: Increased sintering temperatures enhance the diffusion bonding between reinforcement particles and the matrix, resulting in stronger interfacial connections [197]. This enhancement is particularly significant when the sizes of the particles are minimized, as smaller particles provide a greater surface area for bonding and interaction with the matrix. The research studies indicated that at optimized sintering temperatures, a uniform distribution of nano-sized reinforcements can be attained, which helps avoid clustering and bolsters the mechanical properties of composites [198].

*3. Porosity Reduction:* The reduction of porosity is aided by elevated sintering temperatures, which facilitate improved densification. This phenomenon is especially pronounced when fine particles are employed, as they more effectively occupy voids and encourage uniform packing [199]. Nonetheless, if the temperatures are too high, it may result in grain coarsening and the deterioration of the mechanical properties of composites.

*4. Thermal Stability:* Mg-based materials with smaller reinforcement particles frequently demonstrate enhanced thermal stability, which is attributed to their capacity to prevent grain boundary movement [200]. However, this stability is essential for preserving the mechanical integrity of composites when subjected to different sintering conditions.

Mg-based composites promote grain growth, the reduction of porosity, and the distribution of reinforcements, especially in composites that incorporate nano-sized reinforcements at an optimal value of sintering temperature [201]. Further, nano-sized reinforcements can often achieve effective bonding and dispersion at lower sintering temperatures, whereas micron-sized particles might require somewhat elevated temperatures to counteract their reduced surface reactivity.

### 3.7. Sintering Time

Likewise, the sintering time also affects the mechanical characteristics of magnesium and its alloy-based composites. Sintering time improves the bonding strength between the matrix and reinforcing particles in MMCs [202,203,204,205]. Sintering time is critical in reducing the voids or pores in the green compact and provides adequate time to increase the strength of the composites [206,207]. However, the relationship between sintering time and sintering density is also a critical aspect that affects the mechanical properties and structural integrity of the composite. Sintering involves several stages, including initial particle contact, neck formation, and densification [208]. During these stages, sintering time plays a significant role in determining the final density of the material. In the initial stage, particles begin to come into contact, forming small necks. The rate of densification is influenced by the time allowed for these necks to grow [209]. The intermediate stage is crucial for densification, as longer sintering time allows for more effective diffusion processes that reduce porosity and increase density [210]. In the final stage, grain growth can occur, which may affect density if it is not controlled properly, as excessive grain growth can lead to reduced mechanical properties [211]. As sintering time increases, the apparent density of Mg-based composites also increases while porosity decreases. However, the studies showed that apparent density can rise significantly with extended sintering times due to enhanced atomic diffusion leading to pore closure [212,213]. Further, longer sintering time generally correlates with larger grain size and reduced porosity. This occurs because prolonged exposure to heat allows for more complete particle bonding and pore elimination through diffusion processes [214]. Aliuzzaman et al. [212] showed that apparent density increased from 4.649 g/cm³ to 4.724 g/cm³ when the sintering time was extended from 1 h to 5 h. While sintering time is crucial, it often interacts with sintering temperature. A higher sintering temperature combined with a longer sintering time tends to yield higher densities due to enhanced mass transport mechanisms [215]. However, it is essential to balance these parameters since excessive sintering temperature or sintering time can lead to undesirable grain growth.

**Figure 14:**
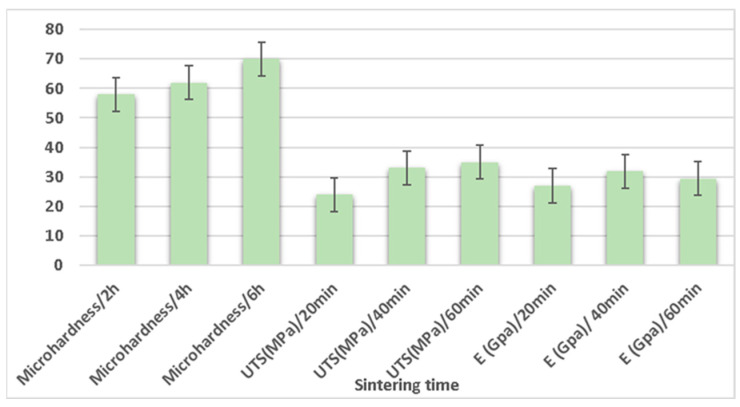
Variation in the mechanical properties of composites via different sintering times in MMCs.

Gunes et al. [216] analyzed the effects of sintering time on the tribological characteristics of magnesium by varying the sintering time (2 h, 4 h, and 6 h) at a sintering temperature of 600 °C. The X-ray diffraction (XRD) image showed MgO, MgO_2_, and Mg compounds after sintering of Mg at 600 °C. The wear rate of magnesium was observed to decrease with an increase in the sintering time. Similarly, the corrosion resistance increased with an increase in sintering time and increased by 36% at a 6 h sintering time compared to a 2 h sintering time. The surface roughness of magnesium was observed to decrease with an increase in sintering time, with a value of 2.47 µm obtained at a 6 h sintering time. The microhardness was observed to be increased with an increase in sintering time, and a value of 75 Hv at a 6 h sintering time was obtained. The continuous reduction in porosity was observed with an increase in the sintering time. Kumar et al. [77] analyzed the effect of sintering time when recycling AZ91 magnesium alloy formed via powder metallurgy by varying the sintering time at values of 1 h, 1.5 h, and 2 h. The theoretical density of the composite was enhanced with an increase in the sintering time. The optimum value to recycle AZ91 magnesium alloy was 2 h. Burke et al. [78] analyzed the effects of sintering time on the mechanical characteristics of magnesium alloy (AZ91) formed via powder metallurgy by varying the sintering time, i.e., 20 min, 40 min, and 60 min. The theoretical density of the magnesium alloy increased with a sintering time of up to 40 min, but beyond that time, there exists a reduction in the theoretical density of the magnesium alloy. The hardness of the magnesium alloy was reduced by increasing the sintering time. The mechanical characteristics, i.e., ultimate tensile strength and elastic modulus, increased with a sintering time of up to 40 min, but beyond that time, there is a reduction in the mechanical characteristics with the increase in sintering time. The influence of sintering time on the mechanical properties of the composite is shown in Figure 14.

## 4. Conclusions

This study explores the possibility of magnesium as a replacement for traditional alloys. Magnesium is known to be the lightest metal, with a density of 1.76 g/cc. Due to its inherent high strength and stiffness, magnesium is desirable for use in various sectors, including biomedical, automotive, aerospace, and military. However, in order to overcome its drawbacks, magnesium-based metal matrix composites (Mg-MMCs) have been developed. These materials have been developed in response to the intrinsic difficulties of low wear resistance and poor mechanical properties. To improve the mechanical properties of magnesium composites, it is critical to comprehend the complex interactions among manufacturing, composition, processing methods, and the addition of different reinforcing agents. One of the most critical areas of research is reinforcing functions in magnesium matrix composites and how they affect both the materials’ broader range of uses and the overall mechanical characteristics. A variety of reinforcing agents are taken into consideration, such as fly ash, hydroxyapatite (HAp), silicon carbide (SiC), carbon nanotubes (CNTs), boron carbide (B_4_C), titanium carbide (TiC), graphene nanoparticles (GNP), and aluminum oxide (Al_2_O_3_). After investigating several fabrication methods, it was found that powder metallurgy is the most effective way to create magnesium metal matrix composites because it strikes a good balance between efficiency and adaptability. It is demonstrated that the mechanical properties obtained via the powder metallurgy method depend on several processing variables. These consist of the following: matrix composition, sintering duration, sintering temperature, types and rates of reinforcement, and particle size of reinforcement. The result of these studies is a methodical analysis highlighting the critical role that powder metallurgy parameters play in the mechanical behavior of magnesium composites, providing opportunities for precise control and optimization.

The importance of processing factors in shaping mechanical and wear properties and broadening the application area of magnesium metal matrix composites was critically examined. This study provides significant insights into common reinforcing agents, such as SiC, CNTs, GNPs, B_4_C, and TiC, emphasizing their value in enhancing the corrosion resistance, wear resistance, and overall mechanical performance of composite materials. Carbon nanotubes (CNTs) emerge as the most significant reinforcing agent, exhibiting improved wear, mechanical, and corrosion properties, particularly when the weight percentage is kept below 1%. The amount of reinforcement is a significant component in determining composite porosity and preventing reinforcement agglomeration inside the matrix. Further, reinforcement types help maintain homogeneous dispersion within the matrix material, hence improving the overall qualities of the composite. Furthermore, optimization studies reveal 450 MPa as the ideal compaction pressure for finding a compromise between low porosity and high density in the composite. Both matrix and particle sizes are critical factors that significantly affect mechanical properties by affecting the development of agglomerations in the composite material. It has been shown that the sintering temperature range of 550–600 °C is favorable for improving the diffusion of reinforcing agents and matrix materials, which enhances the characteristics of the composite. Also, the study emphasizes the temporal component; 2 h of sintering time is the ideal amount of time to recycle magnesium alloys, strengthen green compacts, and improve the mechanical and corrosion properties of composites made of magnesium matrix material.

## 5. Future Scope

The current review provides a framework for future studies on metal matrix composites based on magnesium. This analysis clarifies that processing parameter optimization lays the foundation for further investigation and refinement. Research on new reinforcing agents beyond the widely utilized ones, such as SiC, CNTs, GNPs, B_4_C, and TiC, may reveal materials with improved characteristics or those that work better together. It is necessary to investigate the stability and long-term performance of magnesium composites in practical applications to gain an understanding of these materials’ long-term dependability and durability. Furthermore, this review’s emphasis on the scalability and cost-effectiveness of the powder metallurgy approach opens new research directions aimed at improving the viability of large-scale production processes. Future research should focus on the state-of-the-art area of magnesium composite additive manufacturing method advancements, which are consistent with the general developments in the additive manufacturing industry. These works can significantly contribute to the development of metal matrix composites based on magnesium and their applications across a wide range of sectors.

## Figures and Tables

**Table 1:** Comparative study of powder compaction dies.

S. No.	Compaction of Dies	General Specifications	Benefits of Dies	Drawback of Dies
1	**Single-piece single-action dies**	Manufacturing of the mold cavity is performed as a single piece. Movement of the upper punch is allowed in compaction • Compaction is performed at room temperature	Ease of manufacturing Reliability is relatively high.	The difference between the density of the top and bottom end of the green compact is relatively high. Removal of the green compact is quite complicated
2	**Split single-action dies**	Splitting of the mold cavity into two pieces Movement of the upper punch is allowed in compaction Compaction is performed at room temperature.	Removal of the compact is easy. During the removal of the compact, the edges are less deformed.	Reliability is relatively low, and the chances of bolt failure are frequent. Fracture of material in the vertical direction is frequent.
3	**Split double-action dies**	Splitting of the mold cavity into two pieces Movement of lower and upper punches allowed Compaction is performed at room temperature.	Much less difference between the density of the top and bottom end of the green compact Removal of the compact is easy	Manufacturing of dies is more complex than single-action dies. Spring and bolt failure occur on a timely basis Fracture of material in the vertical direction is frequent.
4	**Split double-action elevated temperature dies**	Splitting of mold cavity into two pieces Movement of lower and upper punches allowed A heating coil linked with a die provides an adequate heat supply to the powders Compaction is performed at an elevated temperature	High densification High-strength compact Removal of the compact is easy	Manufacturing of dies is more complex Less reliable
5	**Isostatic compression**	Use of flexible mold Hydro-static state of stress applied on powder. All points are free to move	Isotropic and uniform properties Intricate shapes formed easily.	Manufacturing of dies is more complex Cost is high

**Table 2:** Dataset for experiment design for the preparation of Mg-based composite based on Taguchi L9 orthogonal array [77].

S. No.	Compaction Pressure (MPa)	Sintering Temperature (℃)	Sintering Time (min)	Sample Notation	Microhardness (Hv)	SN Ratio for Hardness
Level 1	350	400	60	S1	38.05	31.607
		450	90	S2	52.4	34.386
		500	120	S3	48.11	33.644
Level 2	400	400	90	S4	55.93	34.952
		450	120	S5	63.85	36.103
		500	60	S6	41.72	32.406
Level 3	450	400	120	S7	68.79	36.750
		450	60	S8	80.8	38.148
		500	90	S9	75.79	37.592

**Table 3:** Variance assessment for the microhardness data.

Source	DF	Adj SS	Adj MS	F-Value	*p*-Value
Compaction Pressure	2	1349.40	674.70	16.18	0.058
Sintering Temperature	2	241.23	120.62	2.89	0.257
Sintering Time	2	108.13	54.07	1.30	0.435
Error	2	83.39	41.69		
Total	8	1782.15			
